# Adherence to lifestyle recommendations among Norwegian cancer survivors and the impact of traditional and complementary medicine use: the Tromsø Study 2015–2016

**DOI:** 10.1186/s12906-023-04123-4

**Published:** 2023-08-19

**Authors:** Kiwumulo Nakandi, Faith O. Benebo, Laila A. Hopstock, Trine Stub, Agnete E. Kristoffersen

**Affiliations:** 1 National Research Center in Complementary and Alternative Medicine (NAFKAM), Faculty of Health Science, Department of Community Medicine, UiT The Arctic University of Norway, Tromsø, N-9037 Norway; 2 Systems Epidemiology, Department of Community Medicine, UiT The Arctic University of Norway, Tromsø, Norway; 3 Department of Health and Care Sciences, UiT The Arctic University of Norway, Tromsø, Norway

**Keywords:** Cancer survivors, Health recommendations, Diet, 5-a-day, Physical activity, BMI, Smoking, alcohol consumption, CAM, Complementary and alternative medicine, T&CM, Traditional and complementary medicine, Traditional Medicine, The Tromsø Study

## Abstract

**Introduction:**

Adherence to healthy lifestyle recommendations has positive effects on cancer outcomes yet adherence is low among cancer survivors. Differences in adherence between women and men, phase of survivorship, and other factors that might increase adherence, like the use of traditional and complementary medicine (T&CM), need to be explored. We aimed to study the adherence to national recommendations for a healthy diet (daily intake of ≥ 5 portions of fruit/vegetables), physical activity (150 min of moderate-intensity or 75 min of high-intensity/week), normal body mass index (BMI) (18.5–24.9 kg/m^2^), non-smoking, and low-risk alcohol consumption (women ≤ 10 g/day, men ≤ 20 g/day) among Norwegian cancer survivors and their associations with sex, the use of T&CM, and survivorship phase.

**Methods:**

We used logistic regression, independent sample t-test, and chi-square test to study self-reported (diet, physical activity, smoking, alcohol consumption) and measured (BMI) adherence in 1530 cancer survivors (40 years and above, participating in the population-based Tromsø Study conducted in 2015–2016 (65% attendance). We dichotomized all assessed lifestyle recommendations (adherence = 1 point, non-adherence = 0 points), and created a score for every recommendation (0–5 points). Adherence to individual lifestyle recommendations and the use of T&CM as well as the phase of survivorship was adjusted for sex, age, income, and living with a partner.

**Results:**

Adherence to recommendations was 7.5% for diet, 85.3% for physical activity, 30.5% for BMI, 89.3% for non-smoking, and 87.6% for alcohol consumption. In total 2.3% adhered to all five recommendations concurrently (mean score 2.96 [SD = 0.86]). Women adhered to more recommendations concurrently compared to men (3.03 [SD = 0.90] vs. 2.89 [SD = 0.80] points respectively, [p = .012]). In total, 31% reported the use of T&CM and there were no differences in adherence to individual lifestyle recommendations or concurrent adherence in overall T&CM use compared to non-use. Users of self-help techniques were more likely to adhere to the recommendations of diet (aOR 2.69, 95% CI 1.45–4.98) and physical activity (aOR 6.26, 95% CI 1.51–25.92). Users of traditional healers and users of more than one T&CM modality were less likely to adhere to the low-risk alcohol consumption recommendation, (aOR 0.32, 95% CI 0.13–0.77, and aOR 0.53, 95% CI 1.08–2.17, respectively) compared to T&CM non-users. Survivors with cancer previously (1162) had higher odds of adhering to the recommendation of diet (aOR 2.66, 95% CI 1.36–5.19) than survivors with cancer presently (n = 368), but not to other recommendations.

**Conclusion:**

The health of cancer survivors can be improved through adherence to lifestyle recommendations, yet our study found partial adherence among survivors in Norway, in accordance with findings from other countries. Although overall T&CM use was not associated with higher adherence to lifestyle recommendations, differences in adherence were seen among individual modalities like the use of self-help techniques and traditional healers. Our results suggest the need for intensified follow-up of lifestyle with attention to male survivors and diet among all survivors throughout the cancer survivorship continuum.

## Introduction

Life expectancy after a cancer diagnosis continues to increase in many parts of the world [1] due to improved screening and treatment, and improved general health [2]. However, cancer survivors remain at risk for recurrence, second primary cancer [2], and long-term or late effects of cancer [3, 4]. A cancer survivor is generally defined as someone diagnosed with cancer, regardless of phase or prognosis of the disease, starting from the time of diagnosis until death from or with cancer [5].

Approximately 30–50% of all cancer cases [6] and approximately 50% of cancer deaths [7] in adults are estimated to be due to unhealthy lifestyles. Thus, cancer outcomes are associated with lifestyle factors such as diet, physical activity, smoking, and alcohol consumption [8, 9]. Healthy lifestyles have been found to reduce the risk of recurrence of some cancers [10, 11]. Concurrent adherence to several healthy lifestyle factors has more benefits than adherence to just one factor [12], like lowered mortality [13] and improved health-related quality of life [14]. A Norwegian study among long-term adolescent and young adult cancer survivors found low adherence to physical activity, BMI and smoking [15].

Sex differences in adherence have been identified with female cancer survivors being more likely to meet dietary recommendations and being within the recommended weight range [16]. Female survivors are however less likely to meet the physical activity recommendations compared to male survivors [15, 16]. Living with a partner might lead to a healthier lifestyle before and after diagnosis [17]. Survivors less than five years post-diagnosis have been reported to adhere more to the recommended lifestyles compared to survivors more than those five years post-diagnosis [9]. Non-adherence to the recommended lifestyle behaviors can be due to physical, psychological, social, cultural, and/or environmental challenges [18–24]. Thus, adherence to an overall healthy lifestyle among cancer survivors varies.

A cancer diagnosis can trigger the use of Traditional and Complementary Medicine (T&CM) [25]. T&CM is an umbrella term that captures practitioner-based and self-care practices that are not considered conventional healthcare of a given country [26, 27]. Among cancer survivors in Norway, self-help practices are the most common non-provider based T&CM and include relaxation techniques (49%), meditation (29%), and yoga (28%), natural remedies like Omega 3/6/9 fatty acids (31%), ginger (20%), green tea (17%), and blueberries/blueberry extract (17%) [28]. The most common provider-based T&CM among cancer survivors in Norway are massage/aromatherapy (19%) and acupuncture (11%) [28]. The use of T&CM among cancer survivors varies from 33 to 90%, with some of the use beginning upon cancer diagnosis [4, 29–31]. T&CM centers around health preservation and maintenance [32] and thus, may motivate healthy lifestyle changes [33]. The use of T&CM has been associated with healthy lifestyle habits among cancer survivors [34, 35]. For example, T&CM was shown to be associated with physical activity and improved diet [36]. Norway had approximately 320 000 cancer survivors at the end of 2021 [2] and up to 79% of the Norwegian cancer survivors report using T&CM [4, 30].

It is not known whether the use of T&CM is associated with higher adherence to lifestyle recommendations among Norwegian cancer survivors. Differences in adherence due to sex and phase of survivorship among the same group also need to be studied further. This may inform future interventions and support cancer survivorship care programs, ultimately improving their overall well-being and quality of life. We aimed to study (i) adherence to the national lifestyle recommendations for diet, physical activity, body mass index (BMI), low-risk alcohol consumption, and smoking among cancer survivors with comparisons by sex, (ii) the association between adherence to the recommendations and the use of T&CM, and (iii) adherence at different phases of survivorship in a Norwegian population.

## Materials and methods

### Study population

The Tromsø Study is a population-based study conducted in Tromsø, the largest municipality in Northern Norway, located above the Arctic Circle. The study includes seven repeated surveys so far, Tromsø1-Tromsø7, conducted between 1974 and 2016 [37]. In the seventh survey, Tromsø7 (2015–2016), all inhabitants of Tromsø municipality aged 40 years and above were invited (n = 32 591,) of which 11 074 women and 10 009 men aged 40–99 years participated (65% attendance) [38]. Data collection included questionnaires and clinical examinations. Tromsø7 was approved by the Regional Committee of Medical and Health Research Ethics North (reference 2014/940) and participants signed a consent form at attendance [38].

### Study sample

For this study, we included participants with self-reported present or previous cancer (n = 1635) and excluded those with missing values on all questions regarding the use of T&CM the preceding 12 months (n = 105), resulting in a sample of 1530 participants (368 with cancer presently and 1162 with cancer previously).

### Socio-demographic characteristics

Registered age was presented as a categorical variable (40–67 and ≥ 68 years) and as a mean. Education level was presented in three categories: primary, secondary, and tertiary. Annual income referred to the household’s total gross income in the previous year. Low income was < NOK 350 000, middle income was NOK 350 000–750 000, and high income equated > NOK 750 000. Living status was assessed through living or not living with a partner.

### Cancer status

Self-reported cancer status was defined by the questionnaire question *“Have you ever had, or do you have cancer?”* with alternatives; *“no”, “yes, presently”* and *“previously, not now”*.

### Utilization of traditional and complementary medicine

T&CM use was defined by the questionnaire question *“Have you during the past 12 months visited”* (1) *an acupuncturist*, (2) *a CAM provider (homeopath, reflexologist, spiritual healer etc.)*, 3)*Traditional healer (helper, “reader” etc.)?*, *“Have you used herbal medicines during the last 12 months?”*, and *“Have you used meditation, yoga, qi gong or Tai Chi as self-treatment during the last 12 months?”* (self-help techniques). The response *“Yes”* to any of the questions qualified one as T&CM user and we created a variable dividing T&CM user between single or multiple modality users. Use of acupuncturists and CAM provider was grouped to create complementary medicine provider use. The new variable consisted of traditional healer only, complementary medicine provider only, herbal medicine only, self-help techniques only, and more than one modality use. Any use of T&CM was aggregated to create a variable for overall T&CM use.

### Lifestyle recommendations

Adherence to diet, physical activity, smoking, and alcohol intake were defined by the Norwegian national recommendations, using self-reported questionnaires. BMI was calculated from body height and weight measured by trained personnel with a Jenix DS-102 scale (DongSahn Jenix, Seoul, Korea) [38]. All assessed lifestyle recommendations were dichotomized so that they either satisfied (adherence) or did not satisfy (non-adherence) the recommendations (Table 1).

#### Diet

A healthy diet is composed of several elements [39] and for this study we explored the dietary indicator adherence to five portions of fruit/vegetables a day (diet henceforth) based by the questionnaire question “*How many portions of fruit and vegetables do you eat per day in general? (A portion could be an apple or a salad bowl.)”*. Consumption of five or more portions a day was defined as adherence, all other responses were non-adherence.

#### Physical activity

The national recommendations for physical activity are a minimum of 150 min of moderate intensity per week or 75 min of high intensity per week [40]. For this study, we used the questionnaire question, “*Describe your exercise and physical exertion in leisure timeover the last year”.* The alternative *“Reading, watching TV/screen or other sedentary activity*” was categorized as non-adherence while the alternatives *“Walking, cycling, or other forms of exercise at least 4 hours a week (including walking or cycling to place of work, Sunday walking etc.)”, “Participation in recreational sports, heavy gardening, snow shoveling etc. at least 4 hours a week”* and *“Participation in hard training or sports competitions, regularly several times a week”* were categorized as adherence.

#### Body mass index

BMI is calculated by body weight in kilograms divided by body height in meters squared (kg/m^2^) and is categorized as: underweight 18.4 or lower, normal weight 18.5–24.9, overweight 25.0–29.9, obesity 30 – ≥40.0 or higher. A BMI between 18.5 and 24.9 kg/m^2^ was defined as adherence to the recommended BMI. All other ranges were defined as non-adherence.

#### Smoking

Avoidance of smoking [41] was assessed through the question *“Do you smoke or have you smoked daily?”* with the alternatives *“Yes, now”, “Yes previously”, “No, never”*. Operationally, adherence was defined as never-smoker or previous smoker.

#### Alcohol consumption

The national recommendation is to limit alcohol consumption to no more than 10 g a day for women and no more than 20 g a day for men [42]. From self-reported alcohol intake (frequency and amount), the daily nutrient intake of alcohol in grams per day (g/day) was calculated using the food database KBS AE14 and KBS software at University of Oslo (KBS version 7.3). Operationally, 0–10 g/day for women and 0–20 g/day of alcohol for men was defined as adherence.

**Table 1:** The definition of adherence to lifestyle recommendations

Component	Recommended	Adherence	Non-adherence
Diet	≥ 5 portions a day of fruit/vegetables	≥ 5 portions a day of fruit/vegetables	< 5 portions a day of fruit/vegetables
Physical Activity	150 min of moderate intensity per week / 75 min of high intensity per week	≥ 150 min of moderate intensity per week / ≥75 min of high intensity per week	< 150 min of moderate intensity per week and < 75 min of high intensity per week
BMI	18.5–24.9 kg/m^2^	18.5–24.9 kg/m^2^	<18.5/≥25 kg/m^2^
Smoking	Avoid tobacco smoking	Non-smoking (Never, former)	Current smoker (daily)
Alcohol consumption	No more than 10 g/day for women and no more than 20 g/day for men	≤ 10 g/day for women and ≤ 20 g/day for men	> 10 g/day for women and > 20 g/day for men

#### Lifestyle recommendation score

**Fig. 1:**
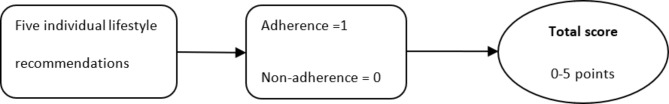
Construction of the total score of concurrent adherences to the assessed lifestyle recommendations

We devised our own scoring system for concurrent lifestyle recommendation adherence by awarding 1 point to any recommendation adhered to and 0 points for non-adherence, Fig. 1. This allowed for a range of 0–5 points, with 5 points indicating adherence to all the assessed lifestyle recommendations.

### Statistical analysis

To ensure sufficient study power and representation of the Norwegian cancer population, we calculated the required sample size considering a margin of error of 5%, a confidence level of 95%, and a heterogeneity of 50%. The minimum sample size needed was determined to be n = 384, considering a population size of 262 884 (cancer survivors in Norway by the end of 2016) [43]. We used descriptive statistics to present the study population characteristics. Categorical variables are presented as numbers and percentages, and continuous variables were described using mean and standard deviation (SD). To evaluate the relationship between lifestyle recommendation adherence and sex (men/women), we dichotomized each lifestyle recommendation, and we performed a Pearson chi-square test. Comparisons of the relationship between mean adherence to concurrent recommendations and sex, phase of survivorship (presently/previously) and use of T&CM (T&CM use/no T&CM use) were performed with an independent sample t-test. To evaluate the relationship between adherence to the lifestyle recommendations and phase of survivorship and use of T&CM, we dichotomized each lifestyle recommendation. Logistic regression was performed with and without adjustment for sex, age, annual income, and living with a partner. The reference category for phase of survivorship was cancer presently. No T&CM use was the reference category for overall T&CM use, as well as for individual T&CM modality use. Unadjusted odds ratios (OR) and adjusted odds ratios (aOR) are presented with 95% confidence intervals (95% CI). A p-value of ≤ 0.05 was considered statistically significant. All analyses were carried out using IBM SPSS version 29.0.

## Results

### Participant characteristics

Participant characteristics are presented in Table 2. The total sample size comprised of 1530 cancer survivors (52.7% women), with mean age 65 years (SD 10), and 73.1% living with a partner. Almost half of the participants reported tertiary education (46.4%) with the majority reporting medium (43.1%) to high income (39.3%). There were three times the number of participants with cancer previously than cancer presently, 75.9% vs. 24.1%, respectively. Thirty one percent (n = 475) used T&CM.

**Table 2:** Basic characteristics of the participants with sex stratification. The Tromsø Study 2015–2016

Characteristic	Total, N = 1530*	%	Women, n = 807	%	Men, n = 723	%	p-value
Age							< 0.001
40–67 years	857	56.0	512	63.4	345	47.7	
Above 67 years	673	44.0	295	36.6	378	52.3	
Mean (SD)	65.16 (10.84)		63.49 (11.21)		67.03 (10.10)		< 0.001
Level of education							0.387
Primary	436	29.2	242	30.6	194	27.6	
Secondary	366	24.5	186	23.5	180	25.6	
Tertiary	693	46.4	363	45.9	330	46.9	
Annual income							< 0.001
Low	268	17.5	171	21.2	97	13.4	
Medium	660	43.1	341	42.3	319	44.1	
High	602	39.3	295	36.6	307	42.5	
Living with a partner							< 0.001
Yes	1069	73.1	487	64.2	582	82.6	
	394	26.9	271	35.8	123	17.4	
Use of T&CM							< 0.001
Yes	475	31.0	295	36.6	180	24.9	
No	1055	69.0	512	63.4	543	75.1	
Survivorship phase							0.001
Presently	368	24.1	154	19.1	214	29.6	
Previously	1162	75.9	653	80.9	509	70.4	

*Total (N) varies due to missing values for some variables

### Overall adherence to the lifestyle recommendations

**Fig. 2:**
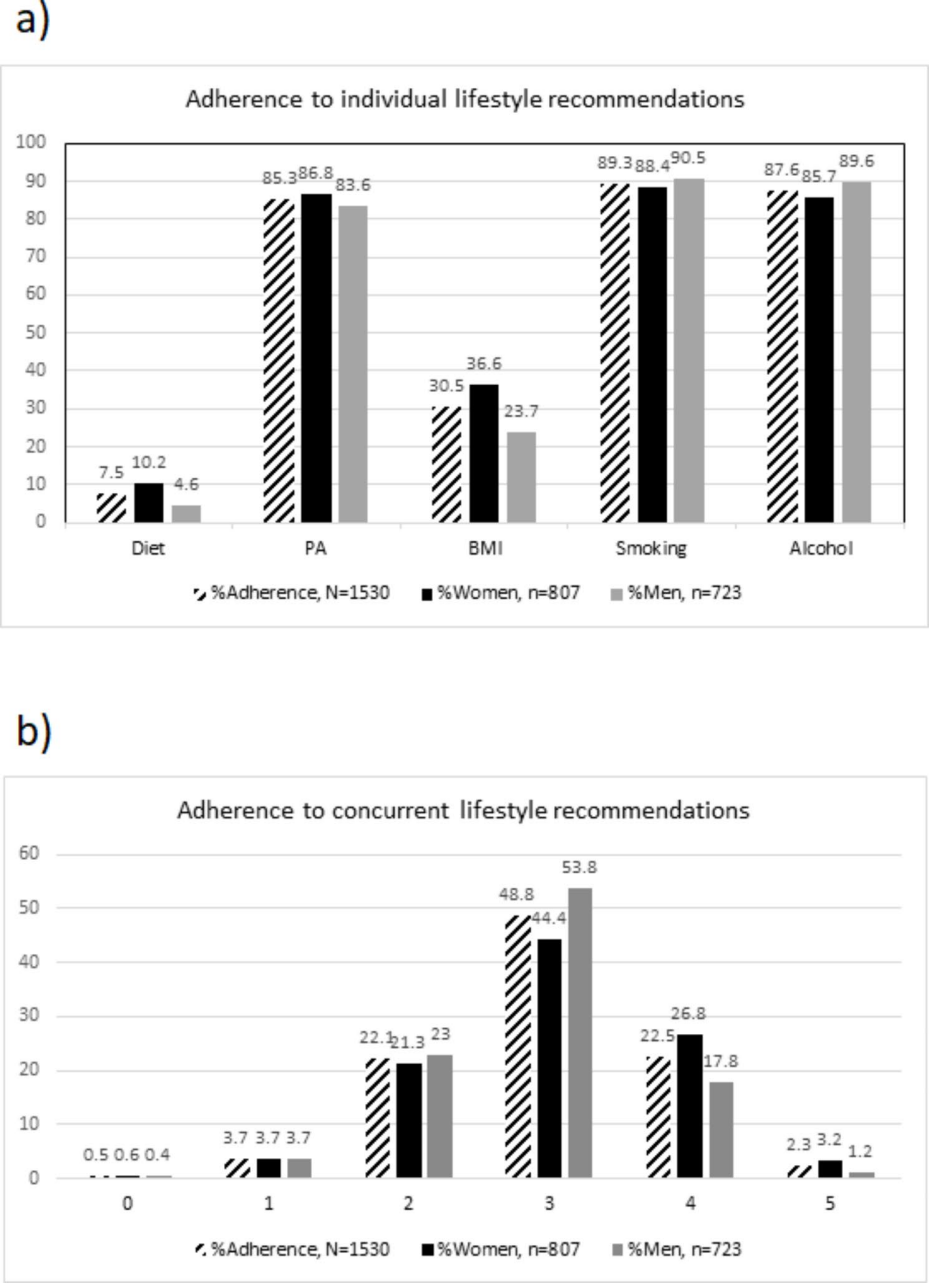
**a)** Reported adherence to the lifestyle recommendations among all cancer survivors. The Tromsø Study 2015–2016. Diet = Five portions of fruit/vegetables a day, BMI = Body Mass Index, PA = Physical Activity, Smoking = Smoking status, Alcohol = Alcohol consumption. **b)** Reported concurrent adherences among all cancer survivors, score 0–5. The Tromsø Study 2015–2016

In total, adherence to recommendations was 7.5% for diet, 85.3% for physical activity, 30.5% for BMI, 89.3% for non-smoking, and 87.6% for alcohol consumption (Fig. 2. a). Consequently, 48.8% met three out of the five lifestyle recommendations, with 2.3% meeting all five recommendations (Fig. 2. b). The mean score of concurrent adherences was 2.96 (SD = 0.86) for all participants. Women adhered to more recommendations than men, mean score 3.03 (SD = 0.90) vs. 2.89 (SD = 0.80) respectively, p = .012. More than twice as many women met the diet recommendation compared to men, 10.2% vs. 4.6% respectively, p < .001. More women met the recommended BMI compared to men, 36.6% vs. 23.7% respectively, p < .001. More men met the low-risk alcohol consumption recommendation compared to women, 89.6% vs. 85.7% respectively, p = .022. No significant sex differences were found in adherence to physical activity and smoking.

### Individual and combined lifestyle recommendations by survivorship phase

Subgroup analysis was undertaken for individual and combined lifestyle recommendations across phase of survivorship. Survivors with cancer previously had higher odds of adhering to the recommendation of diet (OR 3.01, 95% CI 1.60– 5.67), physical activity (OR 1.28, 95% CI 1.00–1.64), and BMI (OR 1.44, 95% CI 1.11–1.88). When adjusted for sex, age, income and living with a partner, survivors with cancer previously had higher odds of adhering to the recommendation of diet (aOR 2.66, 95% CI 1.36–5.19), but not to physical activity, BMI, smoking or alcohol, Table 3.

Survivors with cancer previously had a higher mean score of concurrent adherences compared to cancer presently, 3.02 (SD = 0.87) vs. 2.80 (SD = 0.80), but not a significant level, p = .827.

**Table 3:** Survivorship phase and adherence to lifestyle recommendations. The Tromsø Study 2015–2016

Phase of survivorship	Diet	95% CI	Physical activity	95% CI	BMI	95% CI	Smoking	95% CI	Alcohol	95% CI
Unadjusted OR*										
Cancer presently	1.00	Ref	1.00	Ref	1.00	Ref	1.00	Ref	1.00	Ref
Cancer previously	3.01	1.60–5.67	1.28	1.00–1.64	1.44	1.11–1.88	1.108	0.76–1.61	1.184	0.84–1–67
Adjusted OR**										
Cancer presently	1.00	Ref	1.00	Ref	1.00	Ref	1.00	Ref	1.00	Ref
Cancer previously	2.66	1.36–5.19	1.01	0.71–1.43	1.29	0.97–1.71	1.22	0.82–1.81	1.04	0.71–1.52

*= Unadjusted odds ratio with the lifestyle factor as dependent variable and phase of survivorship as independent variable. **= Adjusted odds ratio for sex, age, annual income and living with a partner

### T&CM use and adherence to lifestyle recommendations

Table 4 shows adjusted odds ratios for adherence to lifestyle recommendations between cancers survivors who reported the use of T&CM (overall, 31%) and no use of T&CM, as well as individual T&CM modalities. We found no statistically significant differences between overall use of T&CM and non-use in adherence to individual lifestyle recommendations. No significant difference was found between mean score of adherences to concurrent lifestyle recommendations between T&CM users (2.99 [SD = 0.83]) and non-T&CM users (2.95 [SD = 0.87], p = .066).

**Table 4:** Adherence to individual recommendations among cancer survivors who use and do not use T&CM. The Tromsø Study 2015–2016

Lifestyle factor	Type of T&CM (n^!^)	OR	95% CI
**Physical activity**	No T&CM (966)	1 (Ref)	
	Overall T&CM (436)	1.33	0.95–1.87
**Physical activity**	No T&CM (966)	1 (Ref)	
	Traditional healer only (24)	1.00	0.33–3.00
	Complementary Providers only (41)	0.74	0.33–1.64
	Herbal medicine only (174)	1.29	0.80–2.07
	Self-help techniques only (80)	6.26	**1.51–25.92**
	More than 1 T&CM modality (117)	1.18	0.67–2.07
**BMI**	No T&CM (1004)	1 (Ref)	
	Overall T&CM (452)	0.85	0.66–1.08
**BMI**	No T&CM (1004)	1 (Ref)	
	Traditional healer only (26)	0.52	0.19–1.14
	Complementary Providers only (42)	0.71	0.35–1.45
	Herbal medicine only (182)	0.77	0.54–1.11
	Self-help techniques only (81)	0.96	0.59–1.56
	More than 1 T&CM modality (121)	1.02	0.68–1.53
**Smoking**	No T&CM (1009)	1 (Ref)	
	Overall T&CM (454)	1.07	0.74–1.55
**Smoking**	No T&CM (1009)	1 (Ref)	
	Traditional healer only (26)	0.72	0.24–2.15
	Complementary Providers only (42)	1.31	0.45–3.80
	Herbal medicine only (184)	1.08	0.63–1.84
	Self-help techniques only (81)	2.76	0.98–7.83
	More than 1 T&CM modality (121)	0.74	0.42–1.28
**Alcohol**	No T&CM (1009)	1 (Ref)	
	Overall T&CM (454)	0.87	0.62–1.22
**Alcohol**	No T&CM (1009)	1 (Ref)	
	Traditional healer only (26)	0.32	**0.13–0.77**
	Complementary Providers only (42)	2.58	0.61–10.9
	Herbal medicine only (184)	1.11	0.67–1.86
	Self-help techniques only (81)	1.66	0.65–4.25
	More than 1 T&CM modality (121)	0.53	**1.08–2.17**
**Diet**	No T&CM (985)	1 (Ref)	
	Overall T&CM (438)	1.12	0.74–1.71
Diet*	No T&CM (985)	1 (Ref)	
	Complementary Providers only (41)	1.30	0.45–3.81
	Herbal medicine only (178)	0.71	0.35–1.46
	Self-help techniques only (80)	2.69	**1.45–4.98**
	More than 1 T&CM modality (115)	0.87	0.40–1.87

Adjusted odds ratio for sex, age, annual income and living with a partner. ^!^ n varies due to missing values. *No traditional healer users adhered to the diet recommendation and were excluded from this analysis

Adherence to individual lifestyle recommendations was analyzed along the use of individual T&CM modalities (traditional healer, complementary providers, herbal medicine, self-help techniques, and more than one T&CM modality) and adjusted for sex, age, annual income and living with a partner. As no users of traditional healers adhered to the diet recommendation, users of traditional healers were excluded from the logistic regression analysis of diet.

Users of self-help techniques were more likely to adhere to the recommendations of diet (aOR 2.69, 95% CI 1.45–4.98) and physical activity (aOR 6.26, 95% CI 1.51–25.92). Users of traditional healers and users of more than one T&CM modality were less likely to adhere to the low-risk alcohol consumption recommendation, (aOR 0.32, 95% CI 0.13–0.77, and aOR 0.53, 95% CI 1.08–2.17, respectively).

## Discussion

Our study shows high adherence to physical activity, non-smoking, and low-risk alcohol consumption recommendations, but low adherence to BMI and even lower adherence to the diet recommendation of five portions of fruit/vegetables a day, with women adhering to more recommendations compared to men. Although overall T&CM use was not associated with increased adherence to the recommended healthy lifestyles, differences in adherence were seen in individual T&CM modalities. The findings of low adherence to diet and BMI are of concern as non-adherence to these factors are associated with poor prognosis, recurrence, and reduced health-related quality of life [6, 7, 10, 11, 13, 14]. Survivors with cancer previously were more likely to adhere to the diet recommendation.

Cross-study comparison is limited due to varying ways to operationalize the adherence to different lifestyle recommendations. Regardless, similar results of high adherence to the lifestyle recommendations of physical activity, non-smoking and low-risk alcohol consumption among cancer survivors have been reported elsewhere [9].

A synergetic effect exists between the modifiable lifestyle recommendations with a linear relationship between the number of lifestyle factors adhered to and health benefits among cancer survivors [12]. In the current study, only 2.3% adhered to all the lifestyle recommendations (mean score: 2.96/5). Studies show that adherence to multiple recommendations is low, but has been improving over time [9, 15]. Emotional struggles and family responsibilities have been reported as barriers for adhering to healthy lifestyles among cancer survivors [18]. Further, frustration of not seeing change in health or body weight has been reported to deter people from adhering to healthy lifestyles as it negatively affects motivation [24]. Views of the lack of impact lifestyle has on cancer outcome, especially following effective anticancer treatment have been reported among cancer survivors [19].

Non-adherence can also be due to an unhealthy lifestyle before a cancer diagnosis that does not change or marginally changes upon diagnosis [44]. Lack of knowledge of the lifestyle recommendations is a contributor to non-adherence. A Norwegian study found that cancer survivors received partial or no information about modifiable lifestyle factors [45], while another study found that 39% of the participants reported getting information about physical activity from health care providers [28]. Furthermore, health literacy is low in some cancer survivors [46] which can contribute to non-adherence.

Female cancer survivors adhered to more recommendations compared to male survivors, especially to diet and BMI. Conversely, men adhered more to the recommendation of low-risk alcohol consumption. As men report more physical symptoms like fatigue and dyspnea, psychological symptoms like depression and anxiety, and lower social functioning [47], this could act as a barrier to adhering to some of the lifestyle recommendations. Our current data does not allow for exploring the reasons for these sex relationships, and future research is needed to investigate these differences.

In addition to the barriers to overall adherence, survivors face individual lifestyle factor-specific barriers.

### Diet

Our study showed that overall daily intake of five portions of fruit/vegetables a day was only 7.5%. Low results of dietary adherence have been reported in several studies along different survivorship phases [15, 48–51]. A Norwegian study showed adherence to the five portions of fruit/vegetables a day recommendation among long-term young adulthood cancer survivors similar to our findings at 8% [15], while another study among lymphoma survivors found adherence as low as 2.2% [52]. Although healthy diet indicators vary widely in studies depending on which factors and how many factors are included (making cross-study comparison limited), the evidence leans towards low adherence to dietary recommendations, including five portions of fruit/vegetables a day [9].

Poor adherence to dietary recommendations is multifactorial. More participants with cancer previously met the five portions of fruit/vegetables a day recommendation compared to survivors with cancer presently in the current study. The reason for these phase-based differences could lie in the challenges of the early phase of survivorship. Cancer and anticancer treatment have been shown to affect smell, taste, appetite, cravings and satiety [53], all of which influence eating habits and dietary patterns of cancer survivors [54, 55]. Other barriers to dietary adherence are gastrointestinal discomfort [55], inadequate information, and lack of advice on culturally relevant healthy diets [18].

There were no differences in adherence to diet between T&CM users and non-users. This can be a result of not distinguishing between T&CM user types. T&CM users can be divided into health-promotion users and symptom-relief users; where health-promotion T&CM users are associated with healthier lifestyle behaviors compared to symptom-relief T&CM users [56]. Norwegian cancer survivors primarily use T&CM to promote quality of life [28], so are more likely to be health-promotion T&CM users. Thus, they would be expected to have healthier lifestyle habits than non-T&CM users. However, Kristoffersen et al. reported T&CM use for treatment intentions in up to 50% among cancer survivors [28]. Mixed users (health-promotion users and symptom-relief users) report less healthy habits compared to health-promotion-only users [56]. The T&CM users of this study might be mixed users, exhibiting no difference in lifestyle behaviors compared to non-T&CM users.

When analyzed along type of T&CM modality, users of self-help techniques were more likely to adhere to the recommended diet. This demonstrates how the grouping of T&CM modalities might suppress nuances in associations of lifestyle adherences. Research on the association between lifestyle and the use of self-help techniques among cancer survivors is limited. However, in the general population users of self-help techniques have been associated with healthier dietary practices compared to non-T&CM users, as well as other forms of T&CM [35]. The tenets of self-help techniques might explain why cancer survivors who use this form of T&CM adhere more to the recommended diet.

### Physical activity

As many as 85% of the participants in this study reported adherence to the assessed levels of physical activity, in accordance with a recent study revealing that 92% of Norwegian cancer survivors had used physical activity to increase quality of life or as a coping strategy related to their cancer [28]. Our finding differs significantly from international studies that reported lower rates of adherence to physical activity recommendations, which ranged from 7 to 41% [57, 58].

Adherence to physical activity post diagnosis is strongly associated with previous physical activity behavior among cancer survivors [59]. Activities like walking could have been common practice before a cancer diagnosis in the current population, contributing to the high numbers of adherence seen in this study. Moreso when lack of facilities/spaces is an identified barrier to engage in physical activity elsewhere [20]. Participants with cancer presently adhered less to the physical activity recommendations in this study and this can be explained by the challenges of the early phase of survivorship. A study among lung cancer survivors undergoing chemotherapy found that physiological factors like fatigue, pain and vomiting limited the duration, intensity, and regularity of physical activity. Psychological factors like anxiety and sociological factors like no social support and feeling useless affected willingness to participate in physical activity [21].

Contrary to other findings of T&CM use being associated with physical activity, we found no associations between physical activity and overall T&CM [35]. However, users of self-help techniques were more likely to adhere to the recommendation compared to no T&CM use or other modalities of T&CM use. The very nature of self-help techniques, like yoga and tai-chi, might explain the high adherence to the physical activity recommendation. Moreso if done in addition to non-T&CM-related activity (like snow shoveling and sports). Users of self-help techniques were also more likely to meet the diet recommendation compared to other T&CM types and non-T&CM use, supporting the findings that users of self-help techniques are motivated to have healthier lifestyles [33, 35].

### BMI

One third of the participants in this study were within the recommended BMI range. Similar and higher findings of adherence to the recommended BMI range have been reported and varies between 34 − 74% [50]. High BMI among cancer survivors has been associated with metabolic disturbances like insulin resistance [60] and anticancer treatment like chemotherapy that can lead to a reduction in physical activity [61]. A recent study showed that in male cancer survivors, lymphedema and depressive symptoms were associated with non-adherence to the recommended BMI [15]. However, high BMI is not a result of individual behaviors and adverse effects alone. It is also linked to obesogenic environments [22]. Furthermore, unwanted weight loss has been reported as a late and long-term effect of a cancer diagnosis and treatment [4] and could also explain low adherence to the recommended BMI among some cancer survivors.

We found no difference in adherence to the recommended BMI and T&CM use and non-use, neither when T&CM was aggregated nor when analyzed as separate T&CM modalities. This contrasts with other findings. Ojukwu et al. found that overweight, but not obese cancer survivors were more likely to use T&CM compared to normal/underweight respondents [31]. The differences in these finding could be multifold. Firstly, 31% of the participants of this study reported the use of T&CM the previous 12 months, compared to the 90% use in the study by Ojukwu et al. Additionally, the current study has a narrower definition of T&CM use. Lastly, the current study dichotomized BMI between normal weight and all other weight classes. This might explain the lack of association between adherence to the recommended BMI and T&CM use among cancer survivors in our study.

### Smoking

The highest adherence we found was for non-smoking, similar to other studies [9]. These findings might be due to the decrease in smoking prevalence in the general population over the years [62] and effectiveness of offering smoking cessation programs to cancer survivors [63]. While adherence to non-smoking is not seen in all the cancer survivors of our study, smokers might have attempted to quit or had cut down. Additionally, some cancer survivors relapse after successful quitting [64]. Concerningly, continued smoking among cancer survivors is related to low perceived disease risk associated with smoking [65] indicating that smoking cessation programs should be emphasized during survivorship care.

### Alcohol

Nearly 90% of the cancer survivors in the current study adhered to the low-risk alcohol consumption recommendations. Although cross-study comparison is limited due to different cut-off values, low-risk alcohol consumption has been reported repeatedly [9, 66]. Some cancer survivors who previously consumed alcohol decrease or stop intake upon diagnosis [67]. This adherence to low-risk alcohol consumption may also be a result of change in taste for alcohol and increased sensitivity to alcohol due to anticancer treatment [68].

There were no differences in adherence to the recommended low-risk alcohol consumption level between overall T&CM users and non-users, as found elsewhere [69]. When segregated along type of T&CM, users of traditional healers and those that used more than one type of T&CM modality were less likely to adhere to the recommended low-risk alcohol consumption level.

Limited research specifically on alcohol consumption among cancer survivors who use traditional healers makes it difficult to compare findings across studies. Our results do not elucidate the directionality of the findings but should be studied further. If the use of traditional healers leads to increased alcohol consumption among cancer survivors, then reasons and the trajectory of the relationship should be identified and addressed. If traditional healers are sought out by cancer survivors to address high alcohol consumption, then traditional healers should be equipped to treat the survivors and conventional health care providers should be made aware of these services.

### Implications

These results identify lifestyle recommendations and associated factors that require more attention for optimum cancer survivorship care. To achieve this, further research should identify facilitators and barriers to adherence to the lifestyle recommendations among Norwegian cancer survivors at the individual, healthcare system, public health, and society level, and addressed accordingly. A comprehensive lifestyle guide and lifestyle specific follow-up consultations with special attention to men and diet throughout the survivorship continuum should be developed.

As data from this study was collected in 2015–2016, newer data should be assessed for the impact on lifestyle of the extended cancer patient pathway (PAKKEFORLØP HJEM FOR PASIENTER MED KREFT) that was introduced in 2022 and intends to follow-up aspects like nutrition [70].

Moreover, future research should aim to analyze T&CM use for each modality individually to better understand the unique mechanisms involved, as aggregating T&CM use may undermine important differences in their associations with examined outcomes.

### Strength and limitations

Our study strengthens the growing knowledge of survivorship care. To our knowledge, it is the first study that looks at lifestyle among Norwegian cancer survivors who use T&CM overall, as well as individual T&CM types. Limitations of this study include group analysis of cancer survivors without considering cancer type or treatment which previous research has shown to affect adherence [71]. The data were self-reported which may be subject to social desirability bias, the desire to be viewed positively by others, which might lead to under- or overestimation of habits [72].

The definition of diet, physical activity, and weight (BMI) are oversimplified in this study. Diet recommendations go beyond 5 portions of fruits and vegetables a day [39]. Using BMI alone can be misleading as it does not specify the composition of the weight (muscle versus fat, subcutaneous versus visceral fat). The study’s questionnaire for assessing physical activity may have resulted in misclassifying individuals who engaged in moderate activity between 150 and 240 min a week as non-adherent, and those who engaged in less than 150 min of moderate activity but were not sedentary as adherent. Never-smokers and previous smokers were grouped together, despite the greater health benefits of never smoking [73]. The study focused on adherence to lifestyle recommendations, not lifestyle-associated risk. Therefore, smoking cessation aligns with the national recommendations and was grouped with never-smokers in this study. Dichotomizing adherence to lifestyle recommendations (adherence vs. non-adherence) may result in loss of information. However, for our study, which did not aim to investigate lifestyle characteristics among cancer survivors, dichotomizing was appropriate. It clearly depicts whether cancer survivors meet the lifestyle recommendations or not.

Another potential weakness of our study is that the prevalence of T&CM use among cancer survivors, which we found to be 31%, appears lower than the findings of a recent Norwegian cross-sectional study. That study reported a T&CM prevalence of 79% among cancer survivors [28]. This discrepancy in findings could introduce a limitation in our study, as it raises questions about the representativeness of our sample and the generalizability of our results to the broader population of cancer survivors in Norway.

## Conclusion

In this population-based sample of women and men with precent and previous cancer, we found that cancer survivors partly adhere to the lifestyle recommendations, and T&CM users did not adhere more to the lifestyle recommendations than non-T&CM users. These results highlight lifestyle recommendations to pay attention to for better cancer survivorship care and provide insights on effect of sex and phase of survivorship and recommended lifestyle adherence. Considering these results and the introduction of the extended cancer patient pathway, a follow-up study of adherence to lifestyle recommendations or change in lifestyle behaviors should be done to assess the effect of the program.

## Data Availability

Data cannot be shared publicly because the current study is based on data owned by a third party (The Tromsø Study, Department of Community Medicine, UiT The Arctic University of Norway). Ethical and legal restrictions prevent data from being made publicly available. Bona fide researchers can apply for data from the Tromsø Study. Guidelines on how to access the data are available at the website https://uit.no/research/tromsostudy. All inquiries about the Tromsø Study should be sent by e-mail to tromsous@uit.no.

## Abbreviations

T&CM: Traditional and Complementary Medicine
SD: Standard Deviation
HD: Norwegian Directorate of Health
BMI: Body Mass Index
g/day: Grams per day
Tromsø7: The seventh survey of the Tromsø Study
OR: Odds Ratio
